# Distributed Power Allocation for Sink-Centric Clusters in Multiple Sink Wireless Sensor Networks

**DOI:** 10.3390/s100302003

**Published:** 2010-03-11

**Authors:** Lei Cao, Chen Xu, Wei Shao, Guoan Zhang, Hui Zhou, Qiang Sun, Yuehua Guo

**Affiliations:** 1 Department of Electronic and Information, The University of Nantong, Nantong Jiangsu, China; 2 Department of Sciences, The University of Nantong, Nantong Jiangsu, China

**Keywords:** wireless communication, sensor networks, multiple sink, clustering hierarchy, power control

## Abstract

Due to the battery resource constraints, saving energy is a critical issue in wireless sensor networks, particularly in large sensor networks. One possible solution is to deploy multiple sink nodes simultaneously. Another possible solution is to employ an adaptive clustering hierarchy routing scheme. In this paper, we propose a multiple sink cluster wireless sensor networks scheme which combines the two solutions, and propose an efficient transmission power control scheme for a sink-centric cluster routing protocol in multiple sink wireless sensor networks, denoted as MSCWSNs-PC. It is a distributed, scalable, self-organizing, adaptive system, and the sensor nodes do not require knowledge of the global network and their location. All sinks effectively work out a representative view of a monitored region, after which power control is employed to optimize network topology. The simulations demonstrate the advantages of our new protocol.

## 1. Introduction

In wireless sensor networks (WSNs) [1], a sensor network is composed of a large number of wireless sensors, densely deployed, in the range of a phenomenon to observe, study and monitor it. A sensor is an electronic device which generally combines three main capabilities: the ability to measure and collect data relative to the environment surrounding it, the ability to process these collected data, and the ability to exchange it with other devices. The other devices can be sensor nodes or sinks. A sink is a particular node, generally with no energy limitation, which collects the information resulting from the sensing nodes, processes it and/or sends it to a data concentration center.

In a WSN cluster protocol, the network is randomly divided into several clusters, where each cluster is managed by a cluster head (CH). The sensor nodes transmit data to their cluster heads, which transmit the aggregated data to the base station. Localized clustering can contribute to more scalable behavior as the number of nodes increases, providing improved robustness, and more efficient resource utilization for many distributed sensor coordination tasks [2]. Data aggregation becomes more simple under cluster conditions. Many clustering algorithms exist in the literature (k-means clustering [3], self-organizing maps [4], LEACH (LEACH-c) [5], TEEN [6], PEGASIS [7], *etc.*).

As a novel issue, WSNs with multiple sinks have become a hot research topic. Many research works have focused on how to deploy sink nodes at optimal locations so the networks can cover relatively larger distances [8,9]. Even many mobile sink schemes were proposed [10] in wireless self-organized networks. This suggests that information about each mobile sink’s location be continuously propagated through the sensor field to keep all sensor nodes updated with the direction for forwarding future data reports. Unfortunately frequent location updates from multiple sinks can lead to both excessive drain of sensors’ limited battery power supply and increased collisions in wireless transmissions. Sink mobility brings new challenges to large-scale sensor networking [11]. In our opinion, it is more practical to improve the network’s topology after the sink nodes are deployed by using a routing protocol and power control scheme.

**Figure 1.**
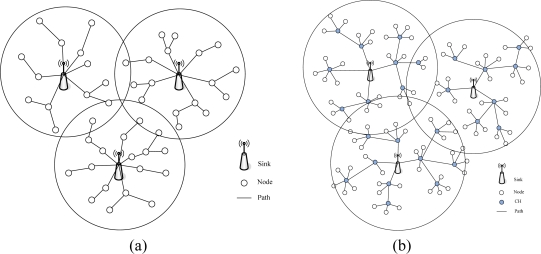
Sink-centric multiple sink cluster WSNs. (a) Sink as cluster head. (b) Non-sink as cluster head.

In this paper, we combine the multiple sink and cluster based routing technology. The MSCWSNs-PC is targeted at multiple sink clustering-based WSNs and is the first power allocation protocol developed for these networks, to our knowledge. The multiple sink cluster WSNs (MSCWSNs) can be simply divided into sink as cluster head and non-sink as cluster head mode. A topology illustration of multiple sink clusters is given in Figure 1. Both types of sink nodes need to negotiate the broadcast radius in order to obtain satisfactory network connectivity and decrease the mutual communication interference.

This paper features the following major contributions:

- It provides a distributed transmission power control algorithm for sink nodes in multiple sink WSNs.
- By using our algorithm, less sensor nodes needs to decide which sink should act as center of local subnetworks.
- The algorithm provides a high network connectivity.
- It is targeted at multiple sink cluster-based wireless sensor networks and is the first protocol developed for these networks, to our knowledge.

In order to design good distributed power allocation protocols for multiple sink wireless microsensor networks, it is important to understand the parameters that are relevant to the sensor applications. While there are many ways in which the properties of a sensor network protocol can be evaluated, we use the following metrics:

### A. Reachability

In multiple sink-centric cluster WSNs, each sensor node will choose at least one sink as its management sink (also denoted as centric sink). It means that the total coverage area by all sinks should be big enough to cover all sub nodes. In this paper, the reachability is defined as the ratio of the number of nodes reached by any one sink to the total nodes deployed in network. Reachability is similar to connectivity.

### B. Power efficiency

The sensor networks should function for as long as possible since it may be inconvenient or impossible to recharge node batteries. Therefore, all aspects of the node, from the hardware to the protocols, must be designed to be extremely energy efficient. In this paper, power efficiency is defined as the mean number of “one-hop” sink (sink to sensor node is one-hop) as each sensor node.

### C. Clustering interference

After the implementation of power allocation, each sink obtains an appropriate transmission power for broadcast operations. Sensor nodes may receive the broadcast packets from more than one “one-hop” sink. These nodes need to decide which sink should to be chosen as centric sink. At this time, clustering interference is taking place.

In this paper, we analyze an efficient multiple sink transmission power control scheme for a sink-centric cluster routing protocol in multiple sink wireless sensor networks. All sinks in the network know their location, and at the same time other sink nodes share their location information. Then every sink decides its communication radius by an absolutely distributed algorithm that uses the location information of the other sink nodes.

The rest of the paper is organized as follows. A summary of related work is presented in Section 2. Section 3 describes the system model of the MSCWSNs-PC protocol. Section 4 describes the design of the MSCWSNs-PC protocol in detail. The performance of MSCWSNs-PC is evaluated in Section 5 and compared with its improved versions using simulation. The paper concludes in Section 6 and some possible improvements to MSCWSNs-PC are pointed out.

## 2. Related Work

Several researchers have proposed routing protocols for utilizing multiple sink nodes [12–16], but only [15,16] proposed a geographic routing. In [15] a grid scenario was assumed, ignoring the routing holes problem, and no details about the real implementation is given. The so-called Greedy Forwarding scheme based routing protocol for multiple sink WSNs is a novel research issue [16].

The advantages of multiple sink wireless sensor networks compared with single sink sensor networks are as follows:

- They are more reliable due to the fact that invalidation of a sink node will drag down the whole network in single sink WSNs.
- Usually there exists a serious node energy bottleneck (around sinks) if a single sink collects reports from too many sensors.
- They relieve the unbalanced energy consumption among sensor networks.
- They avoid mobile sink schemes that result in large energy consumption and serious communication interference [11].
- Multiple sinka reduce payoff of data fusion in very large and complex WSN applications [17].
- They offer more versatile functional applications and communication cooperation. In some applications, different users (sinks) may require different environmental variables (temperature, humidity, light intensity, *etc.*) or data formats (image, sound, video, *etc.*). In this time, all nodes need to cooperate with each other during the communication process.
- In some cluster routing protocols, such as LEACH [5] or PEGASIS [7], each cluster head node needs to communicate with a sink node directly. If only one sink node was deployed, cluster head nodes must work with high transmission power, which not only consumes too much node energy, but also the interference problem of the long distance transmission cannot be ignored.
- In some location-based routing protocols [18] (such as GPSR [19]), the routing holes problem is unavoidable, but it is is expected to be solved effectively in a multiple sink network structure because the sink deployment dispersion will help a sender find a next hop node.
- They provide more real-time data transport of networks, which has a significant effect in multimedia WSNs [20].

At present, the multiple sink sensor networks have been tried in a few applications, such as polar environmental monitoring [21], underwater WSNs [22], *et*c. These applications provide valuable experience for further research on multiple sink networks systems.

In [23], the problem of routing packets in dynamically changing networks is considered, concentrating on two different modes: anycasting and multicasting. In anycasting, a packet has a set of destinations but only has to reach one of them, whereas in multicasting, a packet has a set of destinations and has to reach all of them.

Due to the more balanced energy consumption of clustering-based routing protocols, they are usually employed for large scale WSNs. The LEACH protocol presented in [24] is an elegant solution where clusters are formed to fuse data before transmitting it to the base station. By randomizing the cluster heads chosen to transmit to the base station, LEACH achieves an 8-fold improvement compared to direct transmissions, as measured in terms of when nodes die. But in LEACH all cluster head nodes communicate directly with the base station, which exhausts the nodes far away from base station soon. TEEN considers that cluster heads at increasing levels in the hierarchy need to transmit data over correspondingly larger distances [6]. Combined with the extra computations they perform, they end up consuming energy faster than the other nodes. In order to evenly distribute this consumption, all the nodes take turns in becoming the cluster head for a time interval *T*, called the cluster period. TEEN is well suited for time critical applications and is also quite efficient in terms of energy consumption and response time. PEGASIS (Power-Efficient GAthering in Sensor Information Systems) [7] is a near optimal chain-based protocol that is an improvement over LEACH. In PEGASIS, each node communicates only with a close neighbor and takes turns transmitting to the base station, thus reducing the amount of energy spent per round.

## 3. System Model

### 3.1. Transmission Power Control Algorithms

We assume the WSNs consist of many common sensor nodes and some sink nodes. Each sink node is aware of its own location by using GPS or some other localization mechanism. A sink node broadcasts power control assistant messages (PCAM) to the other sink nodes by one-hop communication. PCAM contains the sender’s location information. We also make some assumptions about the sensor nodes and the underlying network model. For the sink nodes, we assume that all sink nodes can transmit with enough power to reach each other, and that the sink nodes can use a power control scheme to vary the amount of transmission power. Otherwise, each node has the computational power to support different MAC protocols and perform signal processing functions.

In order to obtain satisfactory network connectivity, sink nodes need to negotiate the broadcast radius. A sink node working outside its radio range employs an energy dissipation algorithm to adjust the actual broadcast range.

In [9], the total energy dissipation of node *i* described as:

(1) $$e_{i}^{T}(d)=\alpha d^{n}+\beta$$

where *α,β* ∈ ℛ are real numbers. *β* is the overhead energy, representing the sum of the receiver, sensing and computation energy which is a constant value with varying distance *d*.

A more acknowledged model is proposed in [25]. If node *X* send a packet with power *P_t_*, which is heard by node *Y* with power *P_r_*, the following equation holds:

(2) $$P_{X}=P_{Y}\;{\left(\frac{\lambda }{4\pi d}\right)}^{n}g_{t}\;g_{r}$$

where *λ* is the carrier wavelength, *d* is the distance between the transmitter and the receiver, *n* is the path loss coefficient, and *gt* and gr are the antenna gains at the transmitter and receiver. The typical value of n is 2. Usually, *λ* = *LightVelocity/Frequency* = 0.1 m or so in 2.4 GHz WSNs applications.

### 3.2. Coverage Rate Analysis

**Theorem 1.** Consider a set of two sinks in an a×b rectangle work area *Z*, and a diagonal line *l*. If the two sinks lie in on different sides of *l*, the intersections of the two radio range circles must be outside the rectangle work area, at the same time the corresponding diagonal vertex (in same position direction) must be inside the radio range of the sink, or else only the sink nearer to the centre of rectangle needs to work, the radio radius chooses the distance between the farthest vertex and itself.

**Figure 2.**
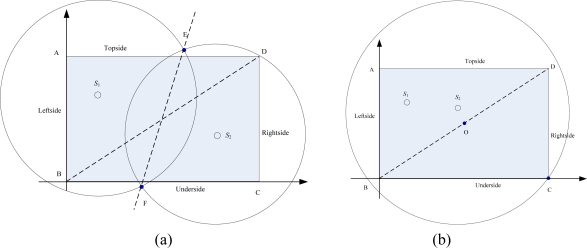
Illustration of WSNs with two sink nodes. (a) Two sinks at different sides; (b) Two sinks on the same side.

**Proof.** Firstly, assume two sinks were deployed at different sides of the diagonal line [see Figure 2(a)], a straight line through the two intersection points E and F, and | *S_2_* *D* |> | *S_2_* *C* |, | *S_1_* *B* |> | *S_1_* *A* |, so the intersection of AEFB and EDCF always contains the rectangle work area ABCD. If the corresponding diagonal vertex (in same position direction, such as *S_2_* and *D*) is inside the radio range of the sink, the radio range of all sinks contains the whole work area. When two sinks are on the same side, the sink nearer to the centre of rectangle uses the distance between the farthest vertex and itself as radio radius, the other three vertices must be in the radio range. It means that the sink has enough transmission power to cover the whole work area. The other sink does not need to work at this time.

Consider a WSN with two sink nodes, as shown in Figure 2. According to the Theorem 1, the radio radius *R_S_i__* in Figure 2(a), *i* ∈ {1,2}, is given by:

(3) $$\{\begin{array}{l} {\left(x_{S_{1}}-x\right)}^{2}+{\left(y_{S_{1}}-y\right)}^{2}=R_{S_{1}}^{2}\\ {\left(x_{S_{2}}-x\right)}^{2}+{\left(y_{S_{2}}-y\right)}^{2}=R_{S_{2}}^{2} \end{array}$$

where (*x*,*y*) is a point in the radio range circle. In order to not only minimize the total transmission power, but also ensure the coverage rate of network, we also define *f* :

(4) $$\begin{array}{l} f=\text{min}\sum_{i=1}^{2}R_{S_{i}}^{n}\\ \text{s.t.}\\ {\left(x_{S_{1}}-x_{A}\right)}^{2}+{\left(y_{S_{1}}-y_{A}\right)}^{2}\leq R_{S_{1}}^{2}\\ {\left(x_{S_{2}}-x_{C}\right)}^{2}+{\left(y_{S_{2}}-y_{C}\right)}^{2}\leq R_{S_{2}}^{2}\\ 0<x_{E},\;x_{F}<a\\ y_{E}\geq b\;\;\;\;\;y_{F}\leq 0 \end{array}$$

where *n* is the path loss coefficient in equation (2). *R_S_i__* in Figure 2(b), *i* ∈ {1,2}, is given by:

(5) $${\left(x_{S_{2}}-x_{C}\right)}^{2}+{\left(y_{S_{2}}-y_{C}\right)}^{2}\leq R_{S_{2}}^{2}$$

In this paper, we only analyse the conditions of two sinks, more sinks, and so forth.

## 4. Power Control For Multiple Sink Cluster WSNs (MSCWSNs-PC)

In this section, we briefly describe the key features of the MSCWSNs-PC algorithms, and some modification algorithms. The protocol discussed includes:

### 4.1. Neighborhood Sink Discovery

**Figure 3.**
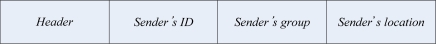
Frame format of *HELLO* packet in MSCWSNs-PC.

In MSCWSNs-PC, only one packet frame format need to be defined, which is denoted as the *HELLO* frame. As shown in Figure 3 the *HELLO* packet carries the *ID* and location information of the sender. After the network initialization, each sink first pre-detects its coarse position in the work area, such as topside, underside, leftside or rightside. Let all sink nodes belong to the corresponding set of *Topside, Underside, Leftside* or *Rightside*. Each sink knows its neighborhood sink by the process of sink discovery communication between them.

### 4.2. Distributed Power Allocation

Assume deployment of *M* sink nodes in an *X* × *Y* region of space, denoted as *S_i_*, and the location be set as (*x_i_*,*y_i_*), *i* ∈ *M*. Let *Z*(*x*,*y*) be the rectangle area, where *x* is the length and *y* is the width. C*S*(*x_i_*,*y_i_*,*r_i_*) is the circle area, where the point (*x_i_*, *y_i_*) acts as the centre and *r_i_* is the radio radius. To ensure the network’s coverage rate up to 100%, the value *r_i_*, *i*∈ *M*, must satisfy the following condition:

(6) $$Z(X,Y)\subset \mathrm{CS}(x_{1},\;y_{1},\;r_{1})\cup \mathrm{CS}(x_{2},\;y_{2},\;r_{2})\cup ...\cup \;\mathrm{CS}(x_{M},\;y_{M},\;r_{M})$$

In [26] the network is represented as the graph *G* = (*N,E*), where *N* is a set of *m*+*n* nodes (containing *m* sinks and *n* sensor nodes), and *S*⊂*N* is the set of sink nodes (with |*S*| = *m*). The set of weighted edges is denoted as *E*, and we note *di;j* as the distance along the shortest path between *i* and *j*. With each sink *k* we associate the *Voronoi cluster V_k_* containing the nodes whose closest sink is *k*. More formally, a *Voronoi cluster V_k_* is defined as:

(7) $$V_{k}=\{i:\underset{j\in S}{\text{min}}\;d_{i,j}=d_{i,k}\}$$

In the *Voronoi* algorithm, each node must be aware of the location of all sink and itself.

**Figure 4.**
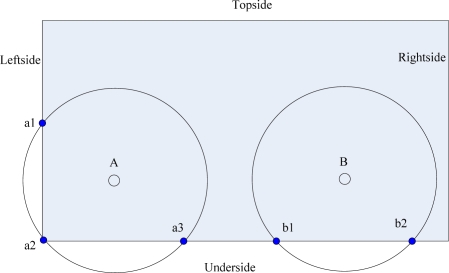
Sink A and B cross with rectangular borders.

In MSCWSNs, firstly each sink pre-detects its coarse position in the work area and the corresponding set of coordinates is (*X_Topside_*, *Y_Topside_*), (*X_Underside_*, *Y_Underside_*), (*X_Leftside_*, *Y_Leftside_*), (*X_Rightside_*, *Y_Rightside_*). The sink number of each group is *nunsink_Topside_, nunsink_Underside_*, *nunsink_Leftside_,* and *nunsink_Rightside_*. Secondly, we make four sequences in a given order, respectively, according to the value of *X_Topside_*, *X_Underside_*, *Y_Leftside_*, and *Y_Rightside_*. Then the sink with the lowest order in each group uses the distance between the corresponding vertices of the rectangle and itself as its radius, then each these sink has three cross points with the boundary of the rectangle, (e.g., node A in Figure 4). The other sink at least have two cross points with the corresponding nearest border by employing an enough transmission power, (e.g., node B in Figure 4). Set a current sink node *w*(*x_w_*,*y_w_*) wants to pre-detect its coarse position in work area.

**Algorithm 1:** sink *w* pre-decide its coarse position

01:	**for all***x_w_, w* ∈ *M*
02:	**if***x_w_*>*Y/*2 **then**
03:	*w* ∈ *Topside*
04:	*nunsink_Topside_* =*nunsink_Topside_+*1
05:	*X_Topside_*(*nunsink_Topside_*)= *x_w_*
06:	*Y_Topside_*(*nunsink_Topsiede_*)= *y_w_*
07:	**else**
08:	*w* ∈ *Underside*
09:	*nunsink_Underside_* =*nunsink_Underside_+*1
10:	*X_Underside_*(*nunsink_Underside_*)= *x_w_*
11:	*Y_Underside_*(*nunsink_Underside_*)= *y_w_*
12:	**endif**
13:	**//**
14:	**if***x_w_*<*X/*2 **then**
15:	*w* ∈ *Leftside*
16:	*nunsink_Leftside_* = *nunsink_Leftside_+*1
17:	*X_Leftside_*(*nunsink_Leftside_*)= *x_w_*
18:	*Y_Leftside_*(*nunsink_Leftside_*)= *y_w_*
19:	**else**
20:	*w* ∈ *Rightside*
21:	*Nunsink_Rightside_* = *Nunsink_Rightside_+*1
22:	*X_Rightside_*(*Nunsink_Rightside_*)= *x_w_*
23:	*Y_Rightside_*(*Nunsink_Rightside_*)= *y_w_*
24:	**endif**
25:	**endfor**

In our opinion, if the intersection of all circle cut off from the edges contained total rectangle borders, the coverage rate of the rectangle work area would be guaranteed. In this time, the *r_i_*, *i* ∈ *M*, must satisfy as the following conditions:

(8) $$\begin{array}{ll} X\subset (2x_{1})\cup \sqrt{{r_{2}}^{2}-{\left(x_{2}-2x_{1}\right)}^{2}}\cup ...\cup \sqrt{{r_{t}}^{2}-{\left(x_{t}-2x_{t-1}\right)}^{2}} & x_{t}\in X_{\mathrm{Topside}}\\ X\subset (2x_{1})\cup \sqrt{{r_{2}}^{2}-{\left(x_{2}-2x_{1}\right)}^{2}}\cup ...\cup \sqrt{{r_{u}}^{2}-{\left(x_{u}-2x_{u-1}\right)}^{2}} & x_{u}\in X_{\mathrm{Underside}}\\ Y\subset (2y_{1})\cup \sqrt{{r_{2}}^{2}-{\left(y_{2}-2y_{1}\right)}^{2}}\cup ...\cup \sqrt{{r_{l}}^{2}-{\left(y_{l}-2y_{l-1}\right)}^{2}} & y_{l}\in Y_{\mathrm{Leftside}}\\ Y\subset (2y_{1})\cup \sqrt{{r_{2}}^{2}-{\left(y_{2}-2y_{1}\right)}^{2}}\cup ...\cup \sqrt{{r_{r}}^{2}-{\left(y_{r}-2y_{r-1}\right)}^{2}} & y_{r}\in Y_{\mathrm{Rightside}}\; \end{array}$$

**Figure 5.**
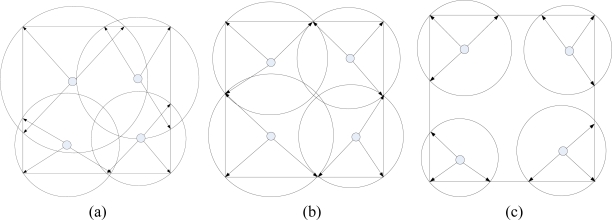
Illustration of radio radius calculation when deploying 4 sink nodes in work area. **(a)** Too large. **(b)** Appropriate. **(c)** Too small.

Figure 5 shows three conditions of radio radius choice, which are too large, appropriate, and too small. It is sure that the appropriate radius is the most desirable. In this article, we attempt to provide a distributed power allocation algorithm by which appropriate radius will be choosen for all sink nodes.

### 4.3. Sink Ordering Orientation

**Figure 6.**
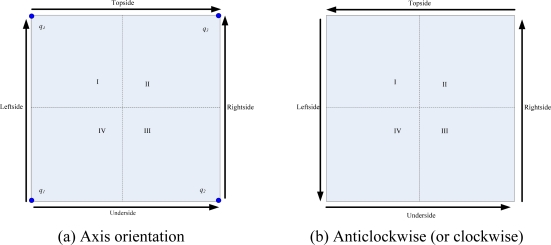
Illustration of radio radius calculation when deploying 4 sink nodes in work area. **(a)**The axis orientation mode. **(b)** the Anticlockwise (or clockwise) mode. Dashed lines refer to the border of quadrant area while solid lines with arrowhead refer to the axis orientation of radio radius calculation.

After all sink nodes knowing their groups as *Topside*, *Underside*, *Leftside*, and *Rightside*, four sequences in given order are obtained respectively according to the value of *X_Topside_*, *X_Underside_*, *Y_Leftside_*, and *Y_Rightside_*. In this paper, two modes of sink ordering orientation are proposed. The axis orientation (See Figure 6 (a)) and the Anticlockwise mode (or clockwise mode, See Figure 6 (b)), they are different in the axis orientation of topside and rightside.

Assume the four vertexs of the objective area are *q_1_*(0,0), *q_2_* (*X*,0), *q_3_*(*X,Y*), *q_4_*(0,*Y*). Before implementation of the MSCWSNs-PC, coordinate axis modification must to be done due to each sink to reckon its radio radius according to the local coordinate information.

Coordinates axis modification in axis orientation mode, is

(9) $$\begin{array}{ll} \{\begin{array}{l} x_{i,j}=x_{j}\\ y_{i,j}=Y-y_{j} \end{array}\;\;\;i\in M,j\in {\mathrm{numsink}}_{\mathrm{Topside}} & \{\begin{array}{l} x_{i,j}=x_{j}\\ y_{i,j}=y_{j} \end{array}\;\;\;\;\;\;\;\;\;\;i\in M,j\in {\mathrm{numsink}}_{\mathrm{underside}}\\ \{\begin{array}{l} x_{i,j}=x_{j}\\ y_{i,j}=y_{j} \end{array}\;\;\;\;\;\;\;\;i\in M,j\in {\mathrm{numsink}}_{\mathrm{Leftside}} & \{\begin{array}{l} x_{i,j}=X-x_{j}\\ y_{i,j}=y_{j} \end{array}\;\;\;i\in M,j\in {\mathrm{numsink}}_{\mathrm{Rightside}} \end{array}$$

While the coordinates axis modification in anticlockwise (or clockwise) orientation mode is

(10) $$\begin{array}{ll} \{\begin{array}{l} x_{i,j}=X-x_{j}\\ y_{i,j}=Y-y_{j} \end{array}\;\;\;i\in M,j\in {\mathrm{numsink}}_{\mathrm{Topside}} & \{\begin{array}{l} x_{i,j}=x_{j}\\ y_{i,j}=y_{j} \end{array}\;\;\;\;\;\;\;\;\;\;i\in M,j\in {\mathrm{numsink}}_{\mathrm{underside}}\\ \{\begin{array}{l} x_{i,j}=x_{j}\\ y_{i,j}=Y-y_{j} \end{array}\;\;\;\;\;\;\;\;i\in M,j\in {\mathrm{numsink}}_{\mathrm{Leftside}} & \{\begin{array}{l} x_{i,j}=X-x_{j}\\ y_{i,j}=y_{j} \end{array}\;\;\;i\in M,j\in {\mathrm{numsink}}_{\mathrm{Rightside}} \end{array}$$

### 4.4. Most significant bit sink radius correction (MSBSRC)

**Figure 7.**
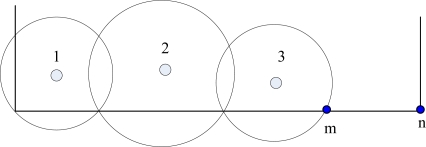
Illustration of border communication void problem.

In original MSCWSNs-PC, sink calculates its radio radius always according to the radio range of previous sink. It often induces a border communication void problem. As shown in Figure 7, the border |*mn*| is defined as border communication void. For this reason, we provide most significant bit sink radius correction (MSBSRC) scheme. After each sink working out its radio radius using original MSCWSNs-PC, then each sink has highest order in each group decides its radius by comparing *R_ij_* and *Distance*(*S_i,j_*, *vert_i_*), *i* ∈ *M*, where *Distance*(*m*,*n*) denotes the distance between *m* and *n*, and *vert_i_* is the vertices set of rectangle work area*, i* ∈ {*Topside, Underside, Leftside, Rightside*} and *j* ∈ { *numsink_Topside_, numsink_Underside_, numsink_Leftside_, numsink_Rightside_*}. Set *w*(*x_w_*,*y_w_*) is a sink node has highest order wants to decide its radius.

**Algorithm 2:** *w* decides its radius by comparing *R_i,j_* and *d*(*S_i,j_*, *vert_i_*)

01:	**for all***w* ,
02:	**//***w*∈ **{ max{**(*X_Topside_*,*Y_Topside_*)**}**, **max{**(*X_Underside_*,*Y_Underside_*)**}**,
03:	**//** **max{**(*X_Leftside_*,*Y_Leftside_*)**}**, **max{**(*X_Rightside_*,*Y_Rightside_*)**} }**
04:	**if***R_i,j_* >= *distance* (*S_i_*, *vert_i_*) **then**
05:	**//***i* ∈ {*Topside*, *Underside*, *Leftside*, *Rightside*}
06:	**//***j* ∈ {*numsink_Topside_, numsink_Underside_*, *numsink_Leftside_, numsink_Rightside_*}
07:	*R_w_*= *R_i,j_*
08:	**else**
09:	*R_w_*= *distance* (*S_i_*, *vert_i_*)
10:	**endif**
11:	**endfor**

*Distance*(*m*,*n*) computes the euclidean distance between nodes *m* and *n* in two dimensions, $\sqrt{{\left(x_{m}-x_{n}\right)}^{2}+{\left(y_{m}-y_{n}\right)}^{2}}$.

### 4.5. Border Constraint Problem (BCP)

**Figure 8.**
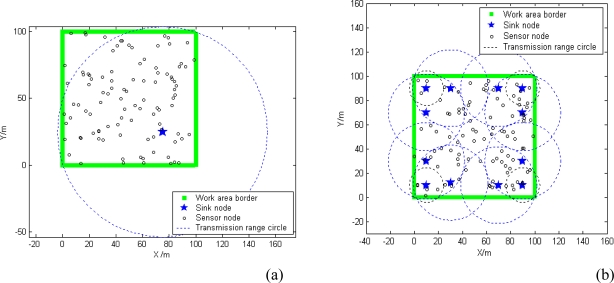
Border constraint problem resulting of sink communication void. (a) sink border group absence. (b) center void.

Although the most significant bit sink radius correction (MSBSRC) scheme is provided, when only a few sink nodes were deployed, the sink border group is absent [see Figure 8(a)] which would result in a communication void. In our opinion, if one or more of the sink groups in *Topside*, *Underside*, *Leftside*, or *Rightside* was empty, this situation will easily produce a sink communication void problem. In addition, the power allocation algorithm is only focused on the borders of the objective area, and the center communication void problem also needs to be considered [see Figure 8(b)]. We denote all of these conditions collectively as the border constraint problem.

For the sink group absence problem, if a sink found that any sink border group was empty, it would chose the distance between the farthest vertex of the border and itself as current radius. In order to solve the center communication void problem, the sink, nearest to the center point of the rectangle in each border group checks if the point has been covered by themselves. If not, they adjust their radio radius equal to the distance between the center point and itself. After these measures, the border constraint problem will be improved greatly.

Let the vertices of the rectangle work area *vert_i_*, *i* ∈ {*I,II,III,IV*}, *I,II,III,IV* denote the quadrants.

**Algorithm 3:** sink *w* decides its radius considering border constraint problem (Anticlockwise orientation mode)

01:	*//***(a) sink border group absence**
02:	**if***i***==**∅ **then**
03:	**for all***w*, *w* ∈ *S_i_*
04:	**//***i* ∈ {*Topside*, *Underside*, *Leftside*, *Rightside*}
05:	**find out the sink *j* which nearest to the corresponding vertex *k* then**
06:	**if** *r_j_*<*distance*(*j*,*k*) **then**
07:	*r_j_*=*distance*(*j*,*k*)
08:	**endif**
09:	**endfor**
10:	**endif**
11:	**// (b) center void**
12:	**for all***w*, *w* ∈ *S_i_*
13:	**find out the sink *m* which nearest to the center vertex *O* then**
14:	*// m* ∈ {*Topside*, *Underside*, *Leftside*, *Rightside*}
15:	**if** *r_m_*<*distance*(*m*,*O*) **then**
16:	*r_m_*=*distance*(*m*,*O*)
17:	**Endif**
18:	**endfor**

### 4.6. MSCWSNs-PC Algorithm Implementation

**Figure 9.**
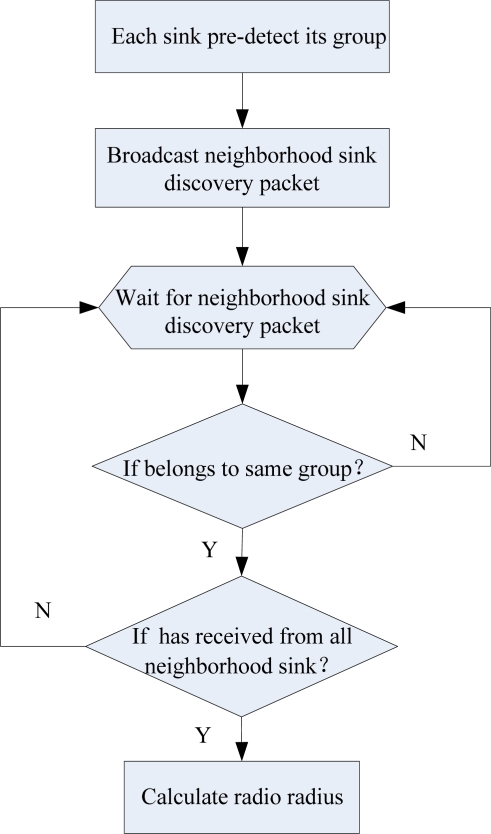
Flow chart for MSCWSNS-PC in each sink.

In this section, we describe the implementation details of MSCWSNs-PC protocol using flowchart. The two main processes are route request and route reply. A flowchart of this distributed power control algorithm is shown in Figure 9.

**Figure 10.**
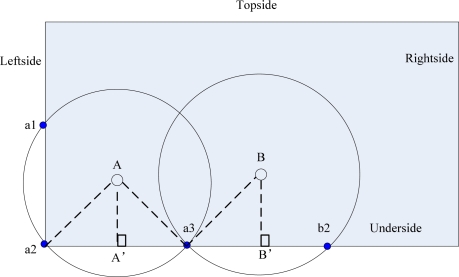
Illustration of radio radius calculation.

Figure 10 gives a simple illustration of a radio radius calculation. For the underside border of the work area, the first sink *A* has the euclidean distance |*Aa_2_*| as its transmission range *R_A,1_*, then sink *B* can calculate *R_B,2_* according to *R_A,1_* by:

(11) $$R_{B,2}=\sqrt{{\left((x_{B}-x_{A})-\sqrt{{R_{A,1}}^{2}-{y_{A}}^{2}}\right)}^{2}+{y_{B}}^{2}}$$

From what has been discussied above, we may safely draw a conclusion that the *R_i,j_*, *i* ∈ *M*, *j* ∈ {*numsink_Topside_, numsink_Underside_*, *numsink_Leftside_, numsink_Rightside_*}, can be calculated by:

(12) $$\{\begin{array}{ll} R_{i,j}=\sqrt{{\left(\left(x_{j}-x_{j-1}\right)-\sqrt{{R_{i,j-1}}^{2}-{y_{j-1}}^{2}}\right)}^{2}+y_{j}^{2}} & i\in M,j\in {\mathrm{numsink}}_{\mathrm{Topside}}\;\mathrm{or}\;j\in {\mathrm{numsink}}_{\mathrm{Underside}}\\ R_{i,j}=\sqrt{{\left(\left(y_{j}-y_{j-1}\right)-\sqrt{{R_{i,j-1}}^{2}-{x_{j-1}}^{2}}\right)}^{2}+x_{j}^{2}} & i\in M,j\in {\mathrm{numsink}}_{\mathrm{Leftside}}\;\mathrm{or}\;j\in {\mathrm{numsink}}_{\mathrm{Rightside}} \end{array}$$

Finally, *r_i_*, *i* ∈ *M*, be calculated by:

(13) $$r_{i}=\text{max}\{R_{i,j}\}\;\;\;\;\;\;i\in M,j\in \{{\mathrm{numsink}}_{\mathrm{Topside}},\;{\mathrm{numsink}}_{\mathrm{underside}},\;{\mathrm{numsink}}_{\mathrm{Leftside}},\;{\mathrm{numsink}}_{\mathrm{Rightside}}\}$$

### 4.7. Created Clusters Communication with Sink Nodes

For the sensor nodes, we assume that all nodes can transmit with enough power to reach the sink if needed, that the nodes can use power control to vary the amount of transmit power, and that each node has the computational power to support different MAC protocols and perform signal processing functions. It means that the created clusters allow energy-efficient communication from the sensors to the sinks, all sensor nodes communicate directly to the sink or they use multi-hop communication in non-sinks as cluster head condition.

### 4.8. Mobility and Transmission Power Maintenance

Sink mobility brings new challenges to routing and data dissemination in large sensor networks. Once any sink has changed its location, all sinks in the border-based sink groups would need to re-build their transmission power. It is important to note that only the sinks in the same group (previously and currently) as the mobile sink would need to take part in transmission power adjustment. It can be seen that our scheme is also suited for mobile networks.

Transmission power maintenance is the process of real-time adjustment of the transmission power for some special conditions, such a a routing failure; at this time alternate routing or a router discovery mechanism need to start for setting up new transmission power allocation for all or some sink nodes. The MSCWSNs-PC protocol since the process of establishment of the radio radius is based on the local connectivity state of adjacent sink nodes, transmission power maintenance can be attributed to the issue of how frequently power control assistant messages (PCAMs) are periodically exchanged.

## 5. Evaluation

This section provides a detailed quantitative analysis comparing the performance of the MSCWSNs-PC protocol using a variety of deployment sink node numbers. We precisely define three metrics for MSCWSNs-PC performance: connectivity, power efficient and clustering interference. The connectivity illustrates the resilience of the algorithm. Because of the rigidly energy constraint in each sensor node, the power efficient is the most important metric for comparison when evaluating WSN protocol schemes. In this paper, power efficiency is denoted as the total transmission power of a sink. The other obvious metric for comparison when evaluating WSN schemes is the clustering interference due to the very high deployment density of sensor nodes. For all of our experiments, sensor nodes are randomly distributed in a 100 × 100 m^2^ rectangular region, the connected sub-network consist of *N* sensor nodes and *M* sink nodes act as the centric nodes.

### 5.1. Deployment Modes of Sink Nodes

Many research works have focused on how to deploy sink nodes at optimal locations, so that the resulting network could cover a relatively larger distance. In our opinion, it is more practical to improve the network’s topology after the sink nodes are deployed using a routing protocol. Transmission power control for MSCWSNs has a significant effect on the percentage of network coverage. In this section, we will illustrate the coverage rate performance of MSCWSNs-PC by using geometric figures. Without loss of generality, we generally not only consider the fixed deployment of sink nodes, but also randomly deployed sinks.

**Figure 11.**
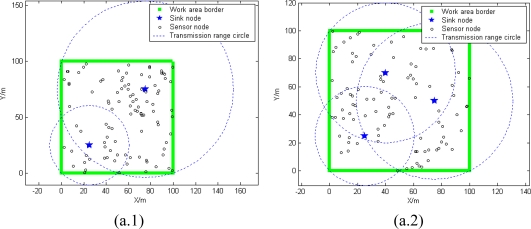

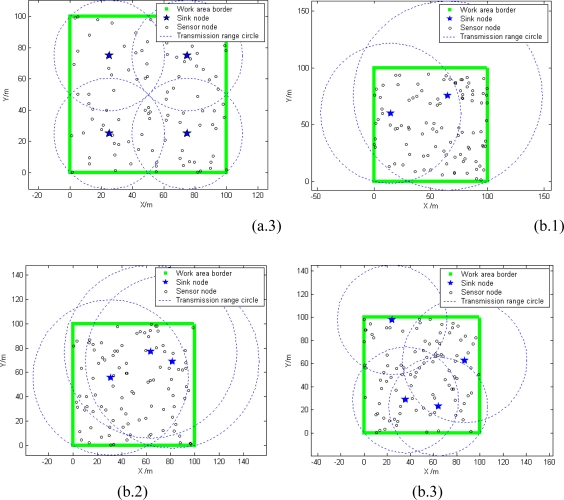
The radio range of sink nodes with power allocation. (a) sink fixed deployment. (b) sink deployment randomly.

Figure 11 shows that whether sinks are fixed or randomly deployed, both conditions can obtain a satisfactory percentage of network coverage. The network is more regular in fixed mode [seen in Figure 11(a)], but more easy to deploy in random mode [as seen in Figure 11(b)].

### 5.2. Sink Orientation to Calculate Radius and Mean Connectivity

**Figure 12.**
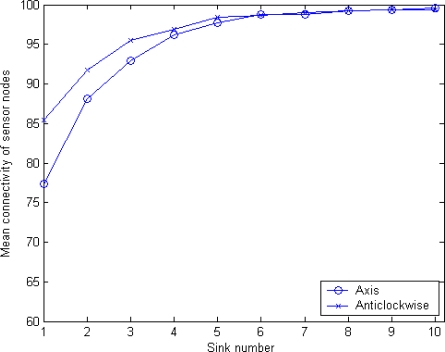
Mean connectivity of MSCWSNs-PC at variety sink number with considering neither MSBSRC nor BCP (mean of 1,000 rounds).

In order to measure the reachability between any sink node and a sensor after execution of our algorithm, we introduce a parameter, *mean connectivity*, which is denoted as *γ*. Mean connectivity is defined as the percentage of the total sensor nodes covered by any sink node in the network. In this section, four schemes described above will be proposed: original MSCWSNs-PC, considering MSBSRC, considering BCP, and considering both of MSBSRC and BCP. For these simulations, two modes of axis ordering orientation are employed simultaneously. In these experiments, generally all results are the statistical average of 1,000 rounds. Figure 12 shows the mean connectivity using the original MSCWSNs-PC at variety sink number. With an increasing number of sinks, the mean connectivity of the network becomes nearly 100%. For the modes of axis ordering orientation, anticlockwise mode brings higher mean connectivity compared to axis mode, with a low sink density of (less than six), but with an increase of the number of sinks the gap between the two lines gradually narrows.

**Figure 13.**
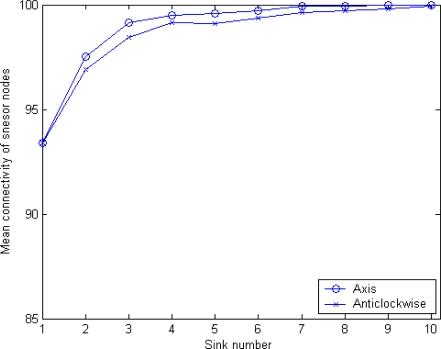
Mean connectivity of MSCWSNs-PC at variety sink number with considering MSBSRC (mean of 1,000 rounds).

Figure 13 shows the relationship between the number of sinks and the mean connectivity of sensor nodes, when only considering MSBSRC. It can be seen that by employing MSBSRC a great improvement in network coverage is achieved. Unlike in original MSCWSNs-PC, axis mode provides higher mean connectivity compared to anticlockwise mode.

**Figure 14.**
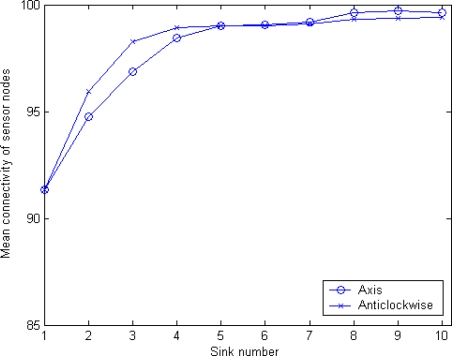
Mean connectivity of MSCWSNs-PC at variety sink number with considering BCP (mean of 1,000 rounds).

Figure 14 shows the relationship between the number of sinks and the mean connectivity of sensor nodes, when only considering BCP. Although BCP results in an obvious increase of mean connectivity compared to the original algorithm, the degree of improvement is smaller compared to the condition only considering MSBSRC. It is noteworthy that with the increase of sinks neither mode of axis ordering orientation is always dominant. The demarcation point is when the number of sinks reaches six, before which anticlockwise mode shows its advantages, while the axis mode is better under other conditions.

**Figure 15.**
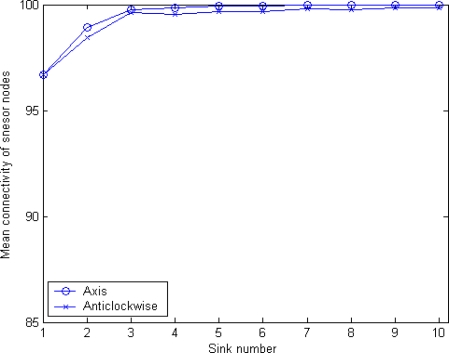
Mean connectivity of MSCWSNs-PC at variety sink number with considering BCP (mean of 1,000 rounds).

Figure 15 shows the mean connectivity result when considering both BCP and MSBSRC schemes. In this condition, the MSCWSNs-PC combines the advantages of the BCP and MSBSRC schemes, and as a result the simulation result is very attractive. Like in Figure 12, axis mode results in higher mean connectivity compared to anticlockwise mode.

### 5.3. Power Efficiency

The sensor networks should function for as long as possible since it may be inconvenient or impossible to recharge node batteries. Therefore, all aspects of the node, from the hardware to the protocols, must be designed to be extremely energy efficient. In this paper, power efficiency is defined as sensor nodes have different number of “one-hop” sink (sink to node is one-hop) when total them with controlled transmission power over maximum transmission power.

Equation (2) gives the relationship of transmission and reception power for wireless devices. Thus, the transmission power of sink node to the sub nodes in a cluster is given by:

(14) $$P_{t}=P_{r}/\left[{\left(\frac{\lambda }{4\pi d}\right)}^{n}g_{t}\;g_{r}\right]$$

Assume has *S* sink in the network, the total transmission power is given by:

(15) $$P_{t}=\sum_{i=1}^{s}P_{t,i}=\sum_{i=1}^{s}P_{r,i}/\left[{\left(\frac{\lambda }{4\pi d}\right)}^{n}g_{t}\;g_{r}\right]=\frac{P_{r,i}\;{\left(4\pi \right)}^{n}}{g_{t}\;g_{r}\;\lambda^{n}}\sum_{i=1}^{s}d^{n}$$

**Figure 16.**
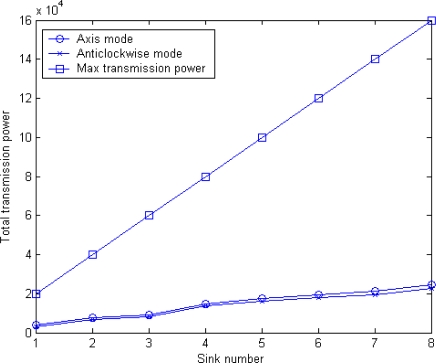
Total transmission power at different sink numbers (n=2).

Figure 16 shows the comparisons of the total transmission power between MSCWSNs-PC and maximum transmission power per round. As shown in Figure 16, MSCWSNs-PC achieves an improvement of several orders of magnitude compared to maximum power transmissions and with the increase of the number of sink the gap between the two lines gradually widens. For the two modes of axis ordering orientation, more total transmission power is needed in axis mode, but the gap between the two modes is very small.

In the well-known energy model of nodes (First Order Radio Model) in [5], the model took into account nodes’ transmitting and receiving energy consumptions, compared to the actual work that node energy model more suitable for constant rate and peer-to-peer links in the mobile node. The literature assumes a simple model for the radio hardware energy dissipation where the transmitter dissipates energy to run the radio electronics and the power amplifier, and the receiver dissipates energy to run the radio electronics. For the experiments described here, both the free space (power loss) and the multipath fading (power loss) channel models were used, depending on the distance between the transmitter and receiver [28]. Power control can be used to invert this loss by appropriately setting the power amplifier - if the distance is less than a threshold, the free space (fs) model is used; otherwise, the multipath (mp) model is used. Thus, to transmit a *k*-bit message a distance, the radio expends:

(16) $$E_{\mathrm{Tx}}\;(k,d)=E_{\mathrm{Tx}-\mathrm{elec}}\;(k)+E_{\mathrm{Tx}-\mathrm{amp}}\;(k,d)=\left\{ \begin{array}{ll} {\mathrm{kE}}_{\mathrm{elec}}+{k\xi }_{\mathrm{fs}}d^{2}, & d<d_{0}\\ {\mathrm{kE}}_{\mathrm{elec}}+{k\xi }_{\mathrm{mp}}d^{4}, & d\geq d_{0} \end{array}\right.$$

and to receive this message, the radio expends:

(17) $$E_{\mathrm{Rx}}\;(k)=E_{\mathrm{Rx}-\mathrm{elec}}\;(k)={\mathrm{kE}}_{\mathrm{elec}}$$

The electronic energy, *E_elec_*, depends on factors such as the digital coding, modulation, filtering, and spread of the signal, whereas the amplifier energy, *ξ_fs_d*^2^ or *ξ_mp_d*^4^, depends on the distance to the receiver and the acceptable bit-error rate. For the experiments described in this paper, the communication energy parameters are set as: *E_elec_* = 50 nJ/bit, *ξ_fs_* = 10 pJ/bit/m^2^, and *ξ_mp_* = 0.0013 pJ/ bit/m^4^. Using the experimental results in [29], the energy for data aggregation is set as *E_DA_* = 5 nJ/ bit/signal.

The first order radio model shows that we should reduce the communication of each sensor node as much as possible. In MSCWSNs-PC, without considering the following process of clustering routing, not only all sinks save power by constraining their radio range, but also all sensor nodes receive a smaller number of sink advertising messages, which greatly saves sensor node battery energy.

### 5.4. Clustering Interference

**Figure 17.**
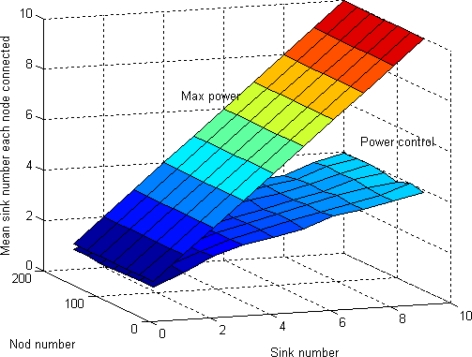
Mean connectivity at variety node number and sink number (100 rounds).

To reduce inter-cluster interference, each cluster in LEACH communicates using TDMA and direct-sequence spread spectrum (DSSS). Each cluster uses a unique spreading code; all the nodes in the cluster transmit their data to the cluster head using this spreading code and the cluster head filters all received energy using this spreading code. This is known as *transmitter-based code assignment* [27], since all transmitters within the cluster use the same code. In this paper, clustering interference is defined as the mean number of sinks a node senses. Wen a node wants to select a sink as its cluster head, the less sink node it hears, the less energy it needs to consume. Figure 17 shows that by using our algorithm, less sensor nodes need to decide which sink they should belong to. The reason is that since all sinks are broadcasting in a more smaller radio range by using MSCWSNs-PC, then less sensor nodes can receive the broadcast packets.

### 5.5. Networks Performance

For our experiments in OPNET, let *M* = {1,2,3,4}, *N* = 28, and four source nodes are randomly deployed in a 100 m × 100 m rectangular region. Radio radius constrained at 20 m. In these experiments, the simulation period *T* is set from 0 to 10 minutes. The data rate created by each source node is 1,000 bits/s.

**Figure 18.**
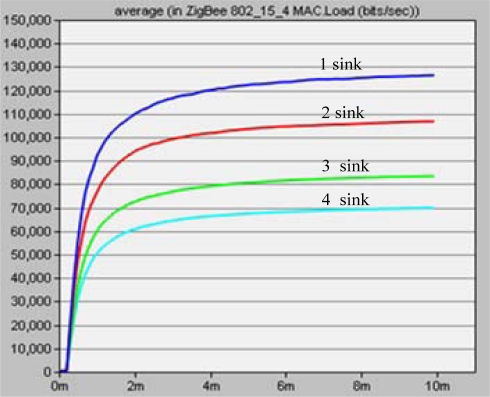
Network load at different sink numbers.

Figure 18 shows network load for different sink numbers. The more sinks deployed, the lower a network load is created. When more sink nodes are deployed, the source node has a chance to choose a nearer sink to transport its data packet to. This results in a significant decrease in the total communication hops, and results in a greatly lower load on the system.

**Figure 19.**
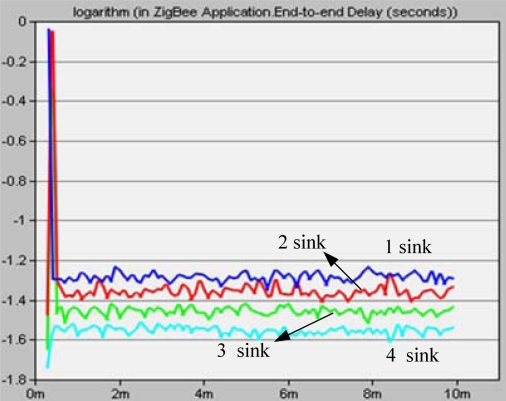
Network load for different sink numbers.

In Figure 19, end-to-end delay of MSCWSNs-PC decreases as sink number increases. The performance of end-to-end delay when using four sink nodes always outperforms in terms of less sinks. In MSCWSNs-PC, more sink nodes result in shorter data transport paths and lower loads which have a significant impact on decreasing networks congestion. Thus, congestion is more likely to happen when less sinks are deployed.

## 6. Conclusions

In this paper, we analyze an efficient multiple sink transmission power control scheme for a sink-centric cluster routing protocol in multiple sink wireless sensor networks. Each sink in the network knows its location, and at the same time the other sink nodes share location information. Then every sink decides its communication radius by a distributed algorithm, which is denoted as MSCWSNs-PC. In a sensor network, our algorithm is executed at each sink node independently. We precisely define three metrics for MSCWSNs-PC performance: connectivity, power efficient and clustering interference. Simulation results confirm that our approach produce a satisfactory network performance. Since the combination of multiple sinks and an adaptive clustering hierarchy routing scheme is a novel topic, more in-depth work needs to be done.
